# Penicillin Treatment Failure in Rabbit Syphilis Due to the Persistence of Treponemes (*Treponema paraluisleporidarum* Ecovar Cuniculus) in the Focus of Infection

**DOI:** 10.3389/fvets.2021.675631

**Published:** 2021-06-17

**Authors:** Vladimír Jekl, Markéta Nováková, Edita Jeklová, Petra Pospíšilová, Jitka Křenová, Martin Faldyna, Miša Škorič, David Šmajs

**Affiliations:** ^1^Jekl and Hauptman Veterinary Clinic, Brno, Czechia; ^2^Department of Pharmacology and Pharmacy, Faculty of Veterinary Medicine, Veterinary University Brno, Brno, Czechia; ^3^Department of Biology, Faculty of Medicine, Masaryk University, Brno, Czechia; ^4^Department of Botany and Zoology, Faculty of SciencVeterinary University Brnoe, Masaryk University, Brno, Czechia; ^5^Department of Infectious Diseases and Preventive Medicine, Veterinary Research Institute, Brno, Czechia; ^6^Department of Pathological Morphology and Parasitology, Faculty of Veterinary Medicine, Veterinary University Brno, Brno, Czechia

**Keywords:** rabbit, syphilis, *Oryctolagus cuniculus*, penicillin, sexually transmitted diseases, *in vivo* propagation, dermatitis

## Abstract

Rabbit venereal spirochetosis, a disease caused by *Treponema paraluisleporidarum* ecovar Cuniculus (TPeC), affects both wild and pet rabbits, and is transmitted sexually and *via* direct contact among animals. Treatment of syphilis in pet rabbits requires administration of antibiotics, including penicillin G, chloramphenicol, or fluoroquinolones. The aim of this work was to elucidate the cause of penicillin treatment failure in rabbit syphilis in a pet rabbit treated in Brno, Czech Republic, and to assess the phylogenetic relatedness of the agent to previously characterized pathogenic treponemes. Following amputation of the infected digits, the second round of penicillin treatment using the same dosage and application route resulted in the disappearance of clinical symptoms within a period of two weeks. The bacterium was successfully isolated from the claws, propagated in three experimental rabbits, and the resulting TPeC strain was designated as Cz-2020. Analysis of four genetic loci revealed that the Cz-2020 strain was similar but also clearly distinct from the only TPeC strain, which had been characterized in detail to date, i.e., the Cuniculi A strain, which was isolated in North America. The strain Cz-2020 represents the first available viable TPeC strain of European origin. DNA sequences encoding five penicillin-binding proteins of the strain Cz-2020 were compared to those of Cuniculi A, which is known to be sensitive to penicillin. The sequences differed in six nucleotides resulting in single amino acid changes in Penicillin-binding protein 1, 2, and 3. Since the second round of treatment was successful, we conclude that the penicillin treatment failure in the first round resulted from the presence of infection foci in claws where treponemes persisted.

## Introduction

Rabbit venereal spirochetosis, caused by *Treponema paraluisleporidarum* ecovar Cuniculus (TPeC), is transmitted sexually and *via* direct contact among infected animals (1). While most cases of rabbit venereal spirochetosis are usually sporadic, local epidemics are known to occur in commercial rabbit farms (2). TPeC infection commonly results in erythema, edema, and/or crusting ulcers in the genitoanal and/or orofacial regions (3). Treatment of syphilis in pet rabbits consists of administration of penicillin G, chloramphenicol, or fluoroquinolones (4). Penicillin is recommended for treatment of syphilis in both animals and humans (5, 6) and thus any indications of resistance to this drug should be documented.

The infecting organism causing rabbit syphilis in farmed rabbits was initially described as *Spirochaeta paralues-cuniculi* (7). Later, rabbit treponemal pathogens were referred to as *Treponema cuniculi* or *T. paraluiscuniculi* (2, 8). In 2013, *Treponema paraluiscuniculi* was reclassified as TPeC (9) and similar, widely spread treponemes causing infections in free living hares (*Lepus europaeus* and *Lepus timidus*) have been identified, i.e., *T. paraluisleporidarum* ecovar Lepus (TPeL) [e.g., (9–11)]. Although being the closest known relatives to the trio of human pathogenic *T. pallidum* subspecies, i.e., *pallidum* (TPA), *pertenue* (TPE), and *endemicum* (TEN), the causative agents of syphilis, yaws and bejel, respectively (10), treponemes infecting lagomorphs are not pathogenic for humans (11). There is, however, an overall high level of clinical manifestation similarities among TPeC and human treponematoses, such as crusty sores in the genitoanal and orofacial areas resembling human yaws or the sexual transmission route typical for human syphilis (12). Rabbits are susceptible not only to rabbit (TPeC) but also to hare (TPeL) and human syphilis. Hence, laboratory rabbits played a fundamental role in propagation and diagnostics of human pathogenic treponemes for decades [e.g., (13, 14)]. While recent advance in *in vitro* cultivation of TPA could lead to decreased need of animals for propagation, rabbit infection model will probably remain vital for a variety of biological experiments with treponemes (15).

TPeC shows 98.1% identity to TPA and TPE on the genetic level (12, 16, 17) with the variability accumulated in several genes (e.g., *tpr* paralogous genes, and TP0136, TP0326, TP0488, TP0548 loci). A part of these variable genes was found to be inactivated in TPeC strain Cuniculi A, which could explain the loss of infectivity to humans, and these genes represent promising candidates for virulence factors of TPA (12). Hence, availability of higher number of treponemal isolates from lagomorphs would be valuable to indentify the true genetic diversity of this bacterial species.

The currently known TPeC isolates/strains include Cuniculi A, Cuniculi H, and Cuniculi M, coming either from the Center for Disease Control and Prevention (the USA), i.e., Cuniculi A, or from a laboratory of S. Lukehart (University of Washington, Seattle, Washington, the USA), i.e., Cuniculi H and M. Minimal data on the genetic diversity within TPeC are based on the three strains isolated in North America. The first difference includes the number of *arp* gene (TP0433) repetitions. While there appear to be 25 repeats of a 60-bp long segment in the *arp* gene in Cuniculi H, only 21 such repeats have been described in the Cuniculi A strain (18). The second difference appears to be a deletion of TP0618 in Cuniculi M and Cuniculi H but not in Cuniculi A (19). To date, only a single strain (Cuniculi A) has been characterized at the genome level (12, 16).

The aim of this study was to determine the cause of penicillin treatment failure in rabbit syphilis in a pet rabbit, determine the phylogenetic position of the agent within the *T. pallidum*/*T. paraluisleporidarum* cluster and establish a viable isolate for further genomic or immunologic analyses. The isolate, designated as Cz-2020, is the first TPeC isolate of European origin.

## Materials and Methods

### A Case of Rabbit Syphilis in a Pet Rabbit

A 10-month-old sexually intact female pet rabbit with dermatological changes in the nasal area was firstly brought to the clinic in December 2019. The lesions had been present for more than 5 months and had already been unsuccessfully treated with crusty skin debridement and antiparasitic therapy (two consecutive treatments of 7 days each, with subcutaneous ivermectin 0.2 mg/kg of body weight). The animal was kept in a single household, as such, cross contamination could be excluded.

**Figure 1:**
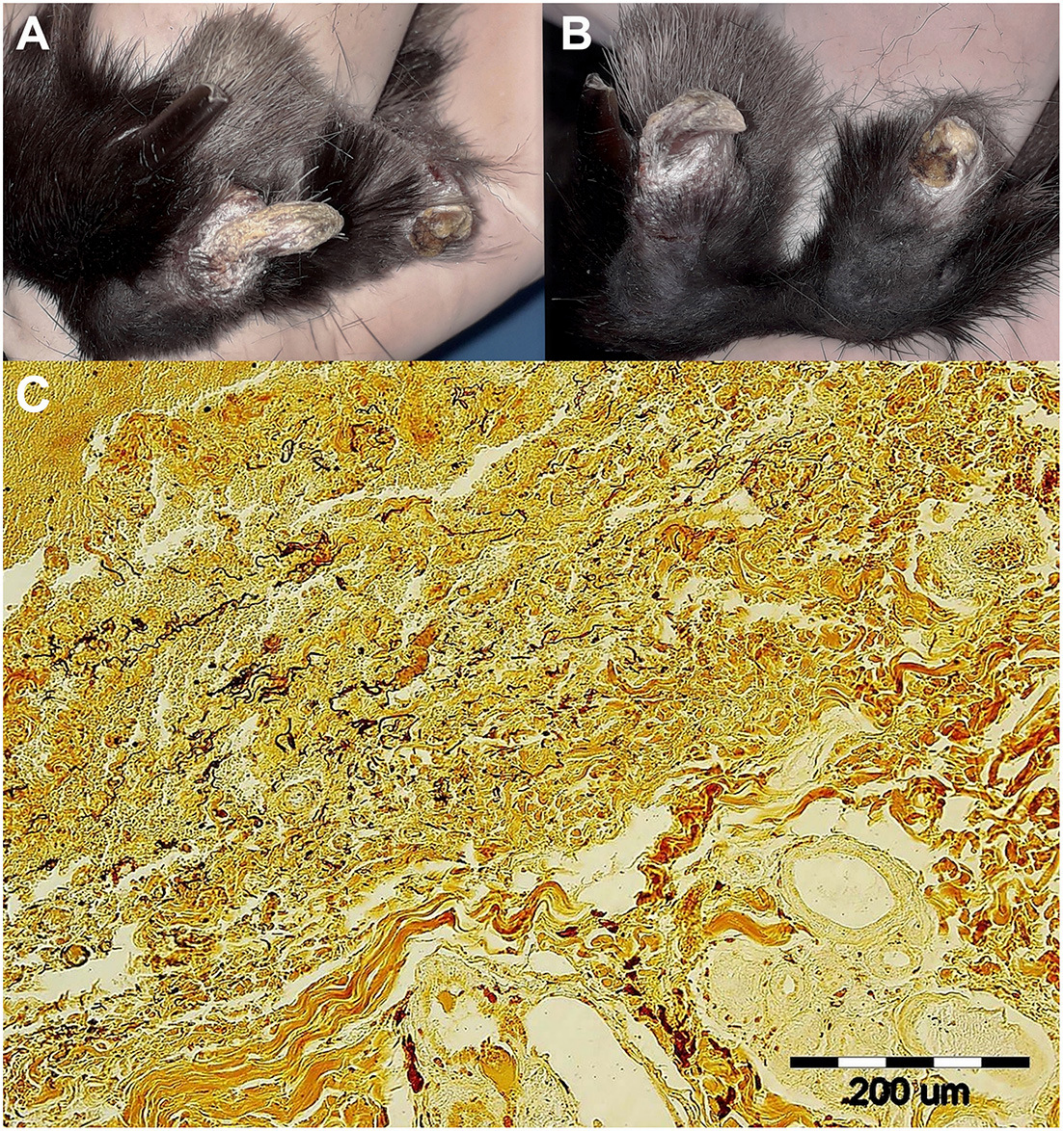
Skin lesions on the right hindlimb of a pet rabbit with syphilis. (**A**, left) Detailed view of healthy (2nd) and syphilis affected (3rd) nail and toe. (**B**, right) Note the fractured and deformed claws/nails on both the 3rd and 4th toes. White scaly lesions were seen on both affected claws/nails, the eponychium, and the distal parts of terminal phalanges. (**C**, bottom) Wartin-Starry silver stain highlights spirochetes in dermis, magnification 200 × . Histopathological examination of the lesion in the area of claw and surrounding connective dermal tissue showed presence of proliferating fibrovascular tissue, moderate mixed inflammatory infiltrate with predominance of lymphocytes, plasma cells, and macrophages, with admixture of lesser number of neutrophils. There was superficial erosion of epithelium, areas of serocellular crusts, focally small hemorrhages and deposits of hemosiderin in dermis. There were no visible bacteria seen in tissue sections stained with HE. Silver staining method (Warthin-Starry) revealed presence of numerous typical spiral and thread-like organisms in epidermis and dermis within the area of inflammatory reaction.

A clinical examination revealed the presence of severe crusty skin changes with areas of proliferation in the area dorsal to the nostrils. Similar skin changes were observed on the skin around the claws and on the skin of the toes on the right hindlimb; no lesions were seen in the anogenital area. All lesions were scaly and white. Skin scrapings and dermatophyte cultures were negative for parasites and dermatophytes. Since the skin lesions were consistent with a diagnosis of rabbit syphilis (4), 14 doses of penicillin G (benzathine/procaine penicillin, 60,000 IU/kg) were administered intramuscularly every 12 h for 7 days. In February 2020, at the 8-week follow-up of the first penicillin treatment, the nasal lesions had completely healed, however, the lesions on the claws and on the toes persisted (Figures 1A,B), which indicated penicillin treatment failure. No other pathologies were found. Clinical material sampled from the claws was used for detection of the pathogen using PCR, dark field microscopy, histopathological staining, and experimental inoculation of rabbits. After sampling, the rabbit claws, as well as the last digit of the right hindfoot, were amputated under general anesthesia due to severe claw changes, including fractures, defective growth, and white scaly areas. At the same time, benzathine/procaine penicillin was administered for a second time, just as it had been during the first round of treatment. A histopathological examination of the amputated tissues revealed the presence of chronic purulent paronychia with reactive fibroblastic changes. Follow-ups at the veterinary clinic at two weeks, 3 and 8 months after the second round of antibiotics, found no disease symptoms, which indicated successful penicillin treatment.

### Collection of Clinical Samples, Dark Field Microscopy, and Histological Staining

Clinical samples were collected from the pet rabbit at the Jekl and Hauptman Veterinary Clinic, Brno, the Czech Republic, in February 2020. The clinical material was taken from a crusty lesion on a claw; it was crushed on a Petri dish, placed in a sterile 1.5 ml Eppendorf tube, and immediately transported, on ice, to the animal facility at the Veterinary Research Institute, Brno, Czech Republic. 1.2 ml of sterile PBS was added and agitated for 25 min at 100 cycles/min to extricate spirochetes according to Lukehart and Marra (20). Five microliters of the suspension were used for dark field microscopy to confirm presence and viability of spirochetes, and to assess their number per ml.

A part of the amputated claws was fixed in 10% buffered neutral formalin, dehydrated, and embedded in paraffin wax. Tissue sections, each prepared on a microtome at a thickness of 4 μm, were stained with Warthin–Starry silver staining according to the manufacturer's instructions (Diapath, Italy).

### Experimental Inoculation

One male New Zealand White rabbit was inoculated in both testes, within 2 h of sampling the pet rabbit, with 500 μl of the suspension extracted from claws per one testicle. The animal was clinically inspected daily, and the inflammatory response (i.e., redness, swelling, and induration of the testes) was monitored. The animal was placed under general anesthesia using medetomidine (0.2 mg/kg IM; Cepetor, Werfft, Germany) and ketamine (15 mg/kg i.m.; Narkamon, Bioveta, Czech Republic), and the testes were aseptically removed, sliced on Petri dishes, each testicle was placed in a 50-ml tube containing 5 ml of sterile PBS and spirochetes were extricated by agitation for 25 min at 100 cycles/min according to Lukehart and Marra (20). The animal was then euthanized using 3 ml IV of the T-61 solution (Intervet International, Netherlands). Laboratory rabbits of the second and third passages were intratesticularly inoculated with suspensions from both testes obtained from the precedent passage (Supplementary Table 1) and daily monitored. Viability and number of treponemes per ml recovered from testicles was assessed by dark field microscopy. Remaining testes suspensions were placed in 1.8 ml Nunc cryogenic tubes [Thermo Fisher Scientific (Waltham, MA, USA)] and stored at −80°C. Housing of all three experimental animals was done in accordance with the Branch Commission for Animal Welfare of the Ministry of Agriculture of the Czech Republic (MZe 2085).

### DNA Isolation

DNA was isolated from 200 μl of sample material in PBS (i.e., material obtained from the infected claw of the pet rabbit or the testes of the laboratory rabbits) as described in Grillová et al. (21), using QIAamp DNA Blood Mini Kit (Qiagen, Hilden, Germany). Isolation was performed within four hours of the samples being received. DNA samples were stored at −20°C prior to PCR analysis.

### PCR Detection

The presence of treponemal DNA was examined using nested PCR detection of TP0105 (*polA*). A list of all primers is shown in Supplementary Tables 2, 3 (22–24). In the first step of the nested PCR, the final volume of the mixture (25 μl) contained 1 μl of DNA, 16.3 μl of water, 5 μl of GXL buffer, 0.095 μl of each primer (100 pmol/μl), and 0.5 μl of Prime STAR GXL polymerase (Takara Bio Europe, France). The touchdown PCR was performed at 94°C for 1 min; 8 cycles: 98°C for 10 s, 68°C for 15 s (−1.0°C per each cycle from cycle 2–8), 68°C for 1 min and 45 s; 35 cycles: 98°C for 10 s, 61°C for 15 s, 68°C for 1 min and 45 s; and 68°C for 7 min. The mixture for the second step was composed of 1 μl of product from the first step, 20.5 μl of water, 2.5 μl of ThermoPol Reaction buffer, 0.5 μl of a 10 mM dNTP mixture, 0.25 μl of each primer (100 pmol/l), 0.1 μl of Taq polymerase (5,000 U/ml; New England BioLabs, Ipswich, MA). PCR was performed at 94°C for 1 min; 40 cycles: 94°C for 30 s, 48°C for 30 s (58°C for *polA*), 72°C for 1 min and 15 s; and 72°C for 7 min. DNA from TPA strain Philadelphia 1 (10 pg/μl) was used as a positive control; ddH_2_O was used as a negative control.

The DNA from the third passage was subjected to molecular typing PCR protocols developed for TPA molecular typing (i.e., TP0548, and TP0705) (25–28) together with TP0488 locus.

Penicillin-binding protein genes, namely TP0500, TP0547, TP0574, and TP0760 were amplified (Supplementary Tables 2, 3). The TP0705 gene, included in the TPA molecular typing, encodes a penicillin-binding protein as well.

### PCR Product Purification, Sequencing, and Sequence Analysis

PCR products were purified using QIAquick PCR Purification Kits (Qiagen, Hilden, Germany) according to the manufacturer's instruction.

Sanger sequencing was performed at GATC Biotech AG (Constance, Germany; Eurofins Genomics Company), and the resulting sequencing reads were assembled and analyzed using Lasergene software (DNASTAR v.7.1.0; Madison, WI, USA).

Sequences of the TP0548 locus, and concatenated sequences of TP0105, TP0488, TP0548, and TP0705 loci were aligned using Muscle algorithm and subjected to maximum likelihood (ML) method and Tamura-Nei model conducted using MEGA v.7.0 (29). Node support was assessed by 1,000 non-parametric bootstrap replicates. The phylogenetic analysis of TP0548 was constructed using strains representing three *T. pallidum* subspecies, the only available TPeC strain Cuniculi A and the only available TPeL strain Z27 A77/78 together with the newly isolated TPeC strain Cz-2020. The ML phylogenetic analysis based on concatenated TP0105–TP0488–TP0548–TP0705 included two *T. pallidum* subspecies and TPeC Cuniculi A and Cz-2020, of which all four loci were available. Sequences of TPeC Cz-2020 generated in the present study are MW323406 (TP0105), MW323407 (TP0488), MW323408 (TP0548), MW323409 (TP0500), MW323410 (TP0547), MW323411 (TP0574), MW323412 (TP0760), and MW323413 (TP0705). Accession numbers of sequences used for phylogenetic analyses are CP004011 and CP004010 for TPA SS14 and Nichols, respectively (30), CP002374 and CP021113 for TPE Samoa D (17) and LMNP-1 (31), respectively, KY120800 for TEN 11q/j (32), and CP002103 for TPeC Cuniculi A (12).

## Results

### Confirmation of Viable Treponemes in the Infected Claw, Isolation, and Propagation of *Treponema paraluisleporidarum* Ecovar Cuniculus Cz-2020 Strain in Experimental Rabbits

**Figure 2:**
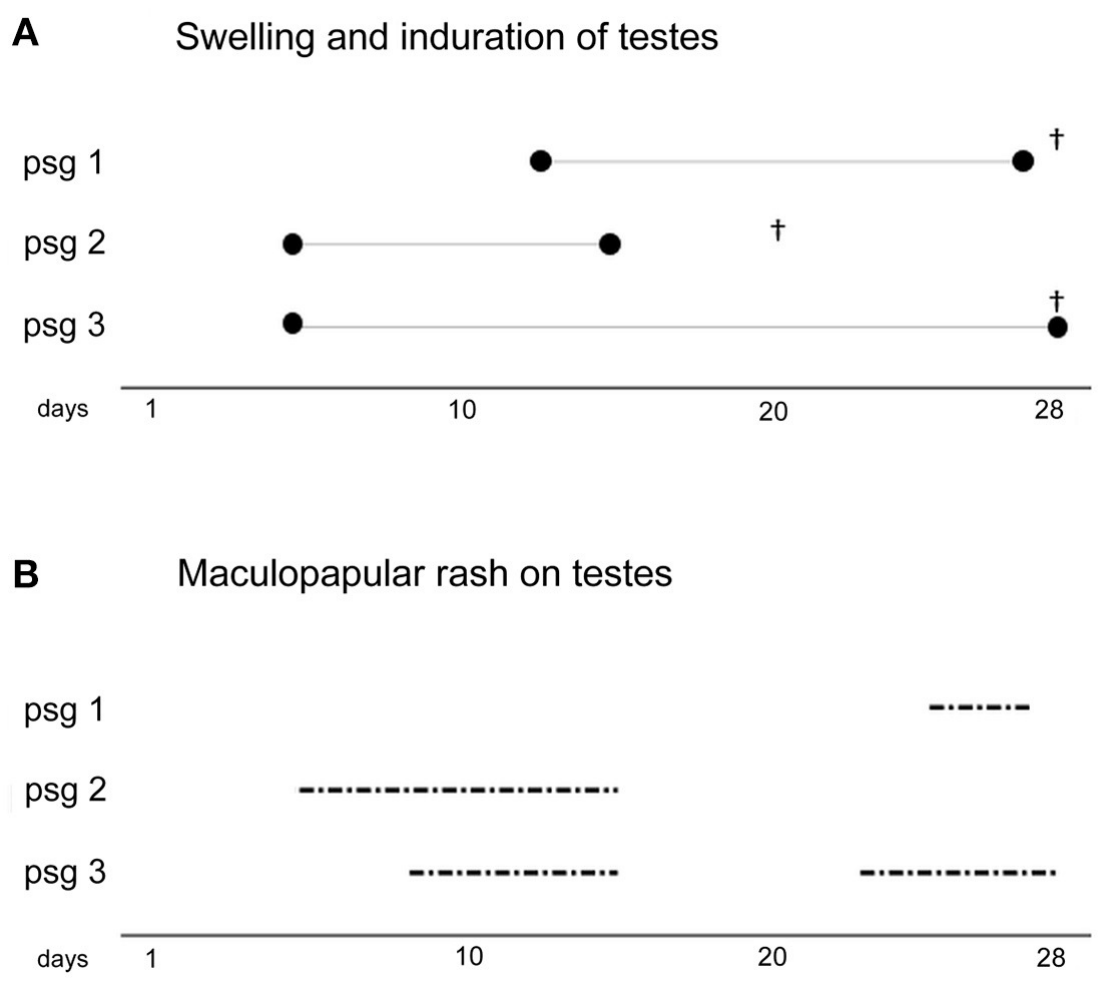
The course of infection in three experimental rabbits inoculated intratesticularly with the TPeC Cz-2020 strain. The cultivation of TPeC Cz-2020 was achieved in three passages (psg), all of which resulted in swelling and induration of the testes and in the appearance of maculopapular rash on the infected testes. (**A**, top) The duration (in days) of inflammation (i.e., swelling and induration of testes) are illustrated by the length of the three line segments delimited by ∙. The day of euthanasia of each animal is depicted by^†^. (**B**, bottom) The duration of rash, for each passage, on scrotal skin is depicted by the dash-dot lines.

The presence of motile, flat, wave-shaped bacteria extracted from the claws was confirmed by dark field microscopy (4.25 × 10^4^/ml) and abundant spirochetes in claw tissue obtained from the pet rabbit were visualized by Warthin Starry histopathological staining (Figure 1C). The initially infected laboratory rabbit was euthanized 28 days post-inoculation (p.i.). Two subsequent passages were performed with a duration of 20 and 28 days p.i. Inoculation doses, numbers of treponemes recovered from all animals and the results from dark field microscopy are summarized in Supplementary Table 1. The course of orchitis is shown in Figure 2. The onset and duration of inflammatory symptoms on testes varied among the three laboratory animals. While the initially infected rabbit developed swelling and induration of testes after 13 days of intratesticular inoculation and maculopapular rash appeared at day 25, swelling and induration of testes started at day 5 in two subsequently inoculated rabbits and rash appeared before day 10. The resulting new TPeC strain was designated Cz-2020.

### Phylogenetic Analysis of TPeC Cz-2020

**Figure 3:**
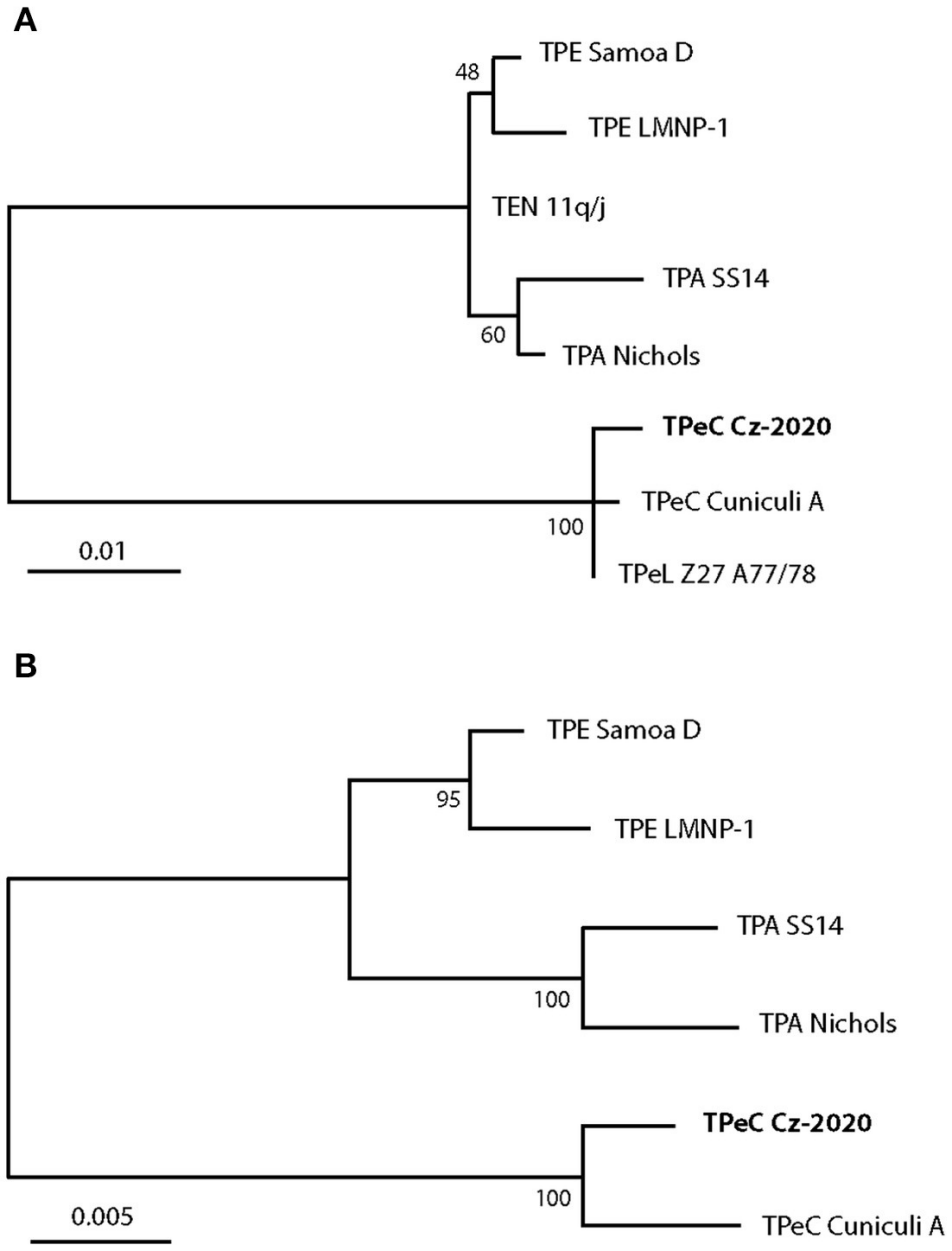
The maximum likelihood phylogenetic analyses of TPeC strain Cz-2020 and selected species of the genus *Treponema*. (**A**, top) The phylogenetic tree is based on the sequence of TP0548 locus showing clustering of TPeC Cz-2020 with TPeC Cuniculi A and with TPeL Z27 A77/78 (the agent of hare syphilis) (9). *Treponema pallidum* ssp. *endemicum* (TEN) 11q/j (32–34) was used because other characterized TEN strains (i.e., Bosnia A and Iraq B) contain a putative recombination event at the TP0548 locus (32). There was a total of 619 positions in the final dataset. All positions with <95% site coverage were eliminated. The scale shows the number of substitutions per site. (**B**, bottom) The phylogenetic tree based on concatenated sequences of TP0105, TP0488, TP0548, and TP0705 loci. TEN subspecies and TPeL are not shown because sequences of TEN 11q/j and TPeL are not available for all these loci. TPeC Cz-2020 is clustered with TPeC Cuniculi A but is still genetically quite distinct. There was a total of 2,945 positions in the final dataset. All positions containing gaps and missing data were eliminated. The scale shows the number of substitutions per site.

A total of eight genomic loci from passage 3 of TPeC Cz-2020 including TP0105 (*polA*), TP0488, TP0500, TP0547, TP0548, TP0574, TP0705, and TP0760 were amplified and sequenced. Both phylogenetic trees (based on TP0548 and on concatenated TP0105–TP0488–TP0548–TP0705 sequences) showed a clear clustering of the TPeC Cz-2020 with other strains isolated from lagomorphs (Figure 3), which confirms that the causative agent of crusty lesions in the pet rabbit was TPeC. The partial sequence (258 nt) of the locus TP0105 of TPeC Cz-2020 was identical to TP0105 of TPeC strain Cuniculi A with query coverage (QC) 100%. The sequence similarities of three remaining loci used for the phylogenetic analysis, i.e., TP0488, TP0548, and TP0705, were 1,018/1,023 (99.51%, QC 100%), 916/950 (96.42%, QC 99.68%), and 2,228/2,232 (99.82%, QC 100%), respectively.

DNA sequences of TP0105 and TP0548 amplified and sequenced from the original claw and from passage 3 were identical, which confirms that TPeC strain Cz-2020 was successfully propagated in three passages.

### Analysis of Genes Encoding Penicillin-Binding Proteins of TPeC Strain Cz-2020

To assess whether there was a sequence-based mechanism for the reduced susceptibility of TPeC Cz-2020 to penicillin, the TP0500, TP0547, TP0574, TP0705, and TP0760 loci, which encode penicillin-binding proteins Pbp-1, LytB, carboxypeptidase 47 kDa, Pbp-2, ad Pbp-3, respectively, were amplified, and the sequences were compared to TPeC Cuniculi A that is sensitive to penicillin. The results are shown in Table 1. Altogether, the sequences of these five genes differed in just six nucleotide positions, resulting in a difference in three amino acids (one amino acid difference in Pbp-1, one in Pbp-2, and one in Pbp-3). Moreover, one of the amino acid replacements (543 H≥R in TP0705) resembled the sequence seen in TPA strains.

**Table 1:** Sequences of TP0500, TP0547, TP0574, TP0705, and TP0760 loci that encode the penicillin-binding proteins in TPeC Cz-2020.

Locus^*^	**Protein**	**Gene position and nucleotide difference relative to TPeC Cuniculi A**	**Number of SNVs/total gene length [nt]**	**Protein position and amino acid difference relative to TPeC Cuniculi A**
TP0500	Pbp-1	55 T → C	2/1875	19 F → L
		711 T → C		No change
TP0547	LytB	No change	0/1131	No change
TP0574	Carboxypeptidase 47 kDa	No change	0/1305	No change
TP0705	Pbp-2	1,628 A → G	1/2655	543 H → R^**^
TP0760	Pbp-3	412 C → T	3/1866	138 R → C
		900 A → G		No change
		1,563 G → A		No change

* *Altogether, 8,832 bp were determined, covering the entire sequences of these loci*.

** *The amino acid change resulted in a residue that is present among TPA strains*.

*The determined sequences were compared to those found in TPeC Cuniculi A*.

## Discussion

Rabbit syphilis is most often seen in rabbit breeding colonies and only occasionally seen in pet rabbits (4, 35). Prevalence of the disease in pet rabbits has been reported in 35.0% (35/100) and 21.3% (26/122) animals in Japan and Korea, respectively (5, 36). In other countries, syphilis in pet rabbits is described only in 0.6% (*n* = 343) (35). Direct inspection of rabbits may lead to underestimation of the prevalence of the disease and suitable serologic assays can be recommended. For example, most of the wild hares infected with TPeL, which is closely related to TPeC, had no visible symptoms (37). However, when treponemal and non-treponemal serological tests were used, the prevalence of TPeL was found to be reaching up to 55.2% among free living brown hares in Europe (38–40). Lesions commonly occur in the anal region, vulva, prepuce, nose, eyelids, and lips (3). Skin changes other than facial or genital lesions have not hitherto been described (4, 41). Interestingly, in the presented case, the syphilitic lesions were also found on the keratinized surface of rabbit claws/nails and the surrounding areas. The syphilis pathogen was confirmed in these novel locations using PCR, histopathological examination, and dark field microscopy of the claw. The spread of the infection to these novel locations is likely a result of habitual face cleaning behaviors.

Treatment of syphilis in pet rabbits consists of antibiotics (4, 42). While treponemes are usually one of the most susceptible bacteria to penicillin, with even small concentrations being bactericidal, they are also one of the most resistant bacteria with respect to the time needed to completely cure the infections (43, 44). In an experimental study performed on rabbits (43), using penicillin G (64,000 IU/kg), a cure was achieved when penicillin G was present, at suitable concentrations, for 6–9 h. Since the plasmatic levels of penicillin G decrease rapidly in rabbits (often within 12 h after intramuscular administration) (45, 46), penicillin G needs to be administered at least twice a day for 7 days.

In this case, the routinely used penicillin treatment described above failed, as demonstrated by the claw/nail and toe lesions that persisted after the first round of penicillin treatment (Figures 1A,B). The second round of penicillin treatment, which was identical to the first, except for being preceded by claw and toe amputations, resulted in the disappearance of clinical symptoms within 2 weeks. In this case, external reinfection can be excluded because the pet rabbit was kept alone in a cage that was carefully disinfected prior to the animal returning from the clinic after the first round of treatment. Moreover, since the second round of penicillin was successful, it is clear that TPeC Cz-2020 is susceptible to penicillin, an observation that is consistent with the minimal genetic differences found in the loci encoding for penicillin-binding proteins. Each of the three mutated penicillin-binding proteins (i.e., Pbp-1, Pbp-2, and Pbp-3) in TPeC Cz-2020 contained only a single amino acid replacement compared to Cuniculi A, which is susceptible to penicillin. Mutated penicillin-binding proteins, i.e., having a lower affinity for β-lactams, are known to encode partial resistance to β-lactam antibiotics (47) and, therefore, each amino acid replacement could potentially encode partial resistance to penicillin. However, the amino acid change in Pbp-2 resulted in a residue that is also present in TPA strains that are known to be fully susceptible to penicillin, suggesting that the amino acid change in Pbp-2 does not encode partial penicillin resistance. Although a partial decrease in treponemal susceptibility due to mutations in Pbp-1 and Pbp-3 cannot be excluded in TPeC Cz-2020, the treatment failure in the case of this pet rabbit case is consistent with penicillin treatment failure due to the presence of the focus of infection. The second round of penicillin treatment, initiated after elimination of the infected claws, led to complete cure of crusty lesions. No disease symptoms were found at 2 weeks, 3 and 8 months follow ups. This study stresses the importance of infection location and the possible reemergence of treponemal infections following antibiotic treatment. Since the cornified surface of claws/nails is avascular, we suggest that penicillin did not reach sufficient concentration in these locations to eliminate the pathogen.

In this study, we isolated a viable new strain of TPeC (i.e., Cz-2020) directly from the claw tissue. We observed that the bacterium retained pathogenicity to rabbits during three subsequent passages. The earlier onset of inflammatory symptoms in passage animals 2 and 3, as shown in Figure 2, may have been caused by one or more of these factors: (i) the initial inoculation of the passage 1 rabbit was nearly 10-times lower than of passage 2 and 3 (Supplementary Table 1); (ii) shorter interval between sampling and inoculation (2 h for passage 1 vs. less than an hour for passage 2 and 3); (iii) the material sampled from the pet rabbit was transported on ice while the testicular extracts were exposed to ambient temperature only.

Routinely uncultivable pathogenic treponemes have highly conserved genomes with a minimal number of variable loci (48). Locus TP0548 was chosen for the phylogenetic analysis in this study since it carries a phylogenetic signal that enables determination on the species and subspecies level (26). It sets apart lagomorph pathogenic species *T. paraluisleporidarum* from human pathogenic *Treponema pallidum* (TP) including all three TPA, TPE, and TEN subspecies (16, 49, 50). In addition, TP0548 also detects variability among strains within subspecies (26). We present that locus TP0548 is substantially variable among all three available *T. paraluisleporidarum* strains (Figure 3A), i.e., the newly isolated TPeC Cz-2020, strain Cuniculi A that also originated from a naturally infected rabbit (51), and TPeL strain Z27 A77/78 that was isolated from *L. europaeus* (9). A larger number of samples obtained from both wild and domestic lagomorphs would help to understand the actual variability and phylogenetic relatedness of *T. paraluisleporidarum* populations infecting animals. In addition to Cz-2020, strain Cuniculi A is the only other fully characterized TPeC strain (12, 16, 51). Two other TPeC strains have been partially described on the genetic level. The first strain is Cuniculi H that appears to differ from Cuniculi A in the number of 60-bp long repeats in the *arp* gene (25 repeats in Cuniculi H vs. 21 in Cuniculi A; (19)). The second example, Cuniculi M, involves deletion of the TP0618 gene, which is also deleted in the Cuniculi H strain, compared to Cuniculi A (18). Although (18) stated that they determined the sequence of the TP0618 locus in Cuniculi A, the whole-genome sequence of Cuniculi A, which was completed in 2011, revealed that the region comprising loci TP0618 through TP0620 was entirely deleted in this strain (12). It is therefore possible that another paralogous genetic region was amplified by the primers designed for TP0618 amplification (18). Therefore, the only genetic difference among the TpeC Cuniculi strains/isolates described so far is the putative difference in the number of *arp* repetitions, in which the exact number of *arp* repetitions in Cuniculi H is still not known precisely (18).

This work provides genetic evidence that TPeC Cz-2020, which is of European origin, is clearly different from TPeC Cuniculi A, which is of North American origin. Moreover, since Cuniculi A was isolated before 1957 (19), the two strains were isolated more than 60 years apart. When a detailed characterization of TpeC Cz-2020 is completed, it will provide valuable insight into the genetic divergence of the causative agent of rabbit syphilis. Since TPeC is not pathogenic to humans, comparative analyses may determine which genes play the crucial role in pathogenesis of human syphilis.

## Data Availability Statement

The datasets presented in this study can be found in online repositories. The names of the repository/repositories and accession number(s) can be found below: NCBI GenBank Nucleotide Accession numbers MW323406, MW323407, MW323408, MW323409, MW323410, MW323411, MW323412, and MW323413.

## Ethics Statement

The animal study was reviewed and approved by MZe 2085. Written informed consent was obtained from the owners for the participation of their animal in this study.

## Author Contributions

VJ conducted treatment of the infected rabbit, noticed a resistance to antibiotic therapy, collected samples, wrote the corresponding part of the sections Materials and Methods, Results and Discussion. MN infected laboratory rabbits, monitored the course of the experimental infection, prepared phylogenetic trees, wrote the corresponding parts of the sections Introduction, Materials and Methods, Results and Discussion. EJ supervised the experimental infection, prepared a part of the section Discussion. PP amplified and analyzed genes for penicillin binding proteins and prepared Table 1. JK isolated DNA, amplified loci for molecular sequence typing system, analyzed Sanger-sequenced traces. MŠ prepared and analyzed histopathological staining. MF and DŠ designated the experiments and wrote initial manuscript. All authors contributed to revisions.

## Conflict of Interest

The authors declare that the research was conducted in the absence of any commercial or financial relationships that could be construed as a potential conflict of interest.
